# A Non-Array Type Cut to Shape Soft Slip Detection Sensor Applicable to Arbitrary Surface

**DOI:** 10.3390/s20216185

**Published:** 2020-10-30

**Authors:** Sung Joon Kim, Seung Ho Lee, Hyungpil Moon, Hyouk Ryeol Choi, Ja Choon Koo

**Affiliations:** School of Mechanical Engineering, Sungkyunkwan University, Suwon 16419, Korea; sjkim314@skku.edu (S.J.K.); smilepop@skku.edu (S.H.L.); hyungpil@me.skku.ac.kr (H.M.); hrchoi@me.skku.ac.kr (H.R.C.)

**Keywords:** tactile sensor, e-skin, slip detection

## Abstract

The presence of a tactile sensor is essential to hold an object and manipulate it without damage. The tactile information helps determine whether an object is stably held. If a tactile sensor is installed at wherever the robot and the object touch, the robot could interact with more objects. In this paper, a skin type slip sensor that can be attached to the surface of a robot with various curvatures is presented. A simple mechanical sensor structure enables the cut and fit of the sensor according to the curvature. The sensor uses a non-array structure and can operate even if a part of the sensor is cut off. The slip was distinguished using a simple vibration signal received from the sensor. The signal is transformed into the time-frequency domain, and the slippage was determined using an artificial neural network. The accuracy of slip detection was compared using four artificial neural network models. In addition, the strengths and weaknesses of each neural network model were analyzed according to the data used for training. As a result, the developed sensor detected slip with an average of 95.73% accuracy at various curvatures and contact points.

## 1. Introduction

The sense of touch is a handy tool when picking up or manipulating objects. Checking the hardness of an object, distinguishing the texture of the surface, and determining the degree of friction are very difficult without the sense of touch. Just as a person acquires information about an object by tactile sense, a robot can also acquire information of an object using a tactile sensor [1]. When a robot repeats routine tasks in a factory, tactile sensors are rarely needed. All objects to be manipulated are in a known state, and tactile sensors have no role. However, as robotic technology advances, robots are now out of the factory to do a dexterous task, and tactile sensors can be utilized at this time [2]. In particular, tactile sensors play a significant role when holding and manipulating unknown objects [3]. When a robot gets out of the existing factory and performs the role of a service robot, it is essential to grasp and manipulate the unknown objects [4].

Conventional tactile sensors measure the vertical force, shear force, torque, and temperature when interacting with an object. Now, sensors that acquire higher-level information using signal processing are being developed [5]. They measure the normal force, shear force, and vibration of the surface to distinguish the material or detect slip on objects. Knowing the slip between an object and a tactile sensor is of great benefit when manipulating an object. If the gripping force increased when the object is slipping, the risk of the robot hand dropping the object could be eliminated [6]. Furthermore, if the robot changes the control method, it can avoid the risk of breaking the object with excessive force [7]. For this reason, research on the slip detection sensor is very active [8].

Various signal processing methods are performed to determine the surface information of an object or whether it slips [5]. Numerous studies have used artificial neural networks (ANN), a type of machine learning. ANN can be used with any sensor as long as it can extract appropriate features from output data. Thanks to these characteristics, it has been used in a variety of tactile sensors. Saccomandi et al. developed a 3 × 3 array force sensor with an error of less than 21 mN by using a feedforward neural network for a tactile sensor using fiber Bragg grating (FBG) [9]. Yuan et al. used convolutional neural networks (CNN) and long short-term memory (LSTM) for GelSight sensors to distinguish objects with different shapes and strengths [10]. Lim et al. used six-layer deep neural networks (DNN) for a piezoelectric array sensor to distinguish the materials of 40 objects with an accuracy of 98.76%. ANN is also widely used for slip detection sensors. Su et al. applied an ANN to the BioTac sensor to detect the slip of an object [11]. They sensed the slip for four objects with different weights and showed an accuracy of more than 80%. Meier et al. used a CNN with a 16 × 16 matrix sensor to determine the slip with 97% accuracy with a delay of less than 10 ms [12]. Li et al. processed the response and vision information of GelSight sensor with DNN to determine the slip with an accuracy of 88.03% in 10 unseen objects [13].

These ANNs make it possible to use simple and inexpensive sensors. Sohn et al. manufactured a very simple MWCNT-PDMS sensor and measured the contact position and force using a DNN [14]. The inexpensive tactile sensor used for disposable was mainly applied in medical robots [15]. However, as the application of tactile sensors has expanded, sensors with a large area are required. Advances in printing technology have helped to make these large area sensors [16]. More sophisticated sensors were developed by the printing [17] or optical shaping [18] of stretchable microstructures. On the contrary, the combination of a large area sensor and ANN showed relative high performance even though their sensors have a simple structure [14,19]. Alternatively, it was used to improve the performance of the sensor [20].

This paper presents a sensor that detects slips effectively by combining an ANN and a simple sensor structure. The developed sensor is designed in a non-array structure with stacked layers, and it can be easily manufactured using inexpensive materials. It is designed to operate even if a part of the sensor is cut under the non-array structure. Thanks to these properties, the developed sensor allows the end-user to directly cut the sensor and attach it to surfaces with arbitrary curvature. The vibration data of the sensor was used to detect the slip. After extracting features from the measured sensor data, the slip was detected using four ANN of different structures. The slip detection performance was tested by attaching it to surfaces with various curvatures to confirm whether the sensor works well on an arbitrary curved surface.

The developed sensor has three contributions: First, it is designed with a very simple mechanical structure and can be produced at a low cost. Most of the existing slip sensors required a complicated production process using an array structure. However, the sensor developed in this paper can be made by simple stacking. Second, the sensor was designed for a cuttable structure. Most tactile sensors only have a limited curvature that fits the sensor. However, the developed sensor can be attached to more complicated surfaces. Finally, we designed a neural network structure suitable for the sensor and compared it. Through this, an optimized ANN structure was derived according to the training data and application surface.

This paper is organized as follows: In Section 2, how the slip sensor was designed and manufactured is presented. In Section 3, how the neural network models were designed to detect slip is described. In Section 4, experimental results show how the developed slip detection sensor works on surfaces with different curvatures. Finally, we summarize the contribution of this paper and conclude the study in Section 5.

## 2. Design of the Slip Detection Sensor

Various types of slip detection sensors that can detect slip have been developed [21]. There are many ways to detect slip using an array of multiple static sensors [11,22]. They analyze how the force distribution on the surface changes and uses vibration information. In addition, to obtain surface information with a higher resolution, optical sensors are sometimes used [23]. These sensors have a complex structure and bigger size, but they can measure slip speed and direction with high accuracy. Furthermore, it is also possible to detect the upcoming slip before the slip occurs [24].

We have developed a tactile skin that can detect slip with a minimal structure. It can be easily manufactured with stacked layers and can be produced at a low cost. The sensor is manufactured in a non-arrayed structure. So, it can be used even if a part of the sensor is cut off. Using this, the sensor that can be attached to any surface has been realized.

### 2.1. Working Principle

Slip refers to the change in relative position between the sensor and the mating surface in contact. When a slip occurs, the contact between the sensor and the mating surface changes, and it makes the magnitude of the frictional force change. This phenomenon is called a stick-slip phenomenon [25]. A change in frictional force causes vibration on the entire sensor. The vibration pattern of a stick-slip phenomenon has characteristics distinguished from disturbances, such as the vibration of a motor. Therefore, the sensor can detect slip by analyzing the surface vibration [26].

When the stick-slip phenomenon occurs, the frictional force generated between the sensor and the object increases and decreases and repeats. This reaction creates a difference in force and acceleration that continuously changes direction on the surface. A sensor that detects slip by measuring such acceleration has been developed [27]. When using an acceleration sensor to detect slip, installing the sensor in a remote location away from the contacting part is possible. It is a great advantage of giving the sensor position freedom. However, even if the object is not in contact, it is greatly affected by disturbing factors such as vibration from the actuator. Using a material having a piezoelectric effect can solve this disadvantage. Since a piezoelectric material generates a voltage by compression and expansion, an output occurs only when a force is applied to both ends of the material. If the object does not contact the piezoelectric sensor, only a tiny output is generated because one side of the sensor becomes a free end. Piezoelectric sensors can be used with this principle to measure vibration only when there is contact.

In the piezoelectric effect, the direction in which the stimulus is mainly felt varies according to the polarized direction of the piezoelectric material. When using a piezoelectric material in the form of a film, the polarized material in the d33 direction is mainly used [28]. This direction is the most sensitive response to vertical forces. However, the vibration caused by the stick-slip phenomenon is most significant in the horizontal direction. In order to convert the horizontal vibration into vertical vibration, a fingerprint structure was employed. The human fingerprint structure plays a role in amplifying the vibration on the surface rather than increasing the frictional force [29]. By simulating such a human fingerprint structure, it can help to detect vibrotactile stimuli well [30]. Also, the fingerprint structure installed on the piezoelectric material protects the sensing element of the sensor.

**Figure 1:**
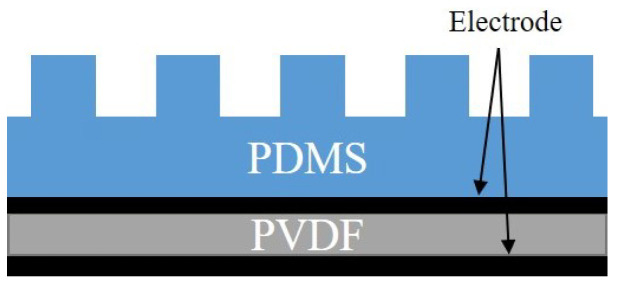
Sensor design.

A sensor with a very simple structure was designed using a piezoelectric material that measures vibration and a fingerprint structure covered on the outside (see Figure 1). It is a form that mimics human skin [31]. The external PDMS structure acts as a fingerprint and amplifies the vibration. The inner PVDF film serves as a sensory receptor and is used as a piezoelectric material to measure vibration. The sensor itself cannot judge whether it is a slip, a change in vertical force, or something else. However, if the sensor can measure the vibration pattern on the surface, it can be used as an input into neural networks that detect slip. Through this process, the designed sensor has the ability to detect slip.

### 2.2. Non-Array Structure

When a tactile sensor measures the force applied to a surface, it is crucial to simultaneously know the force distribution. In the case of a vertical force sensor, the higher the spatial resolution, the more accurate that the force distribution map can be drawn. It is possible to identify the shape of the surface or know where the contact is made using a tactile map with high resolution. However, there is a limit to making denser array to increase resolution. To overcome this limitation, a study to implement a more detailed tactile image with fewer sensing elements has been conducted [32]. Alternatively, a study was conducted to detect the contact position continuously, moving away from the discrete array sensor [33].

It helps improve the accuracy of the slip detection by sensing more data using a high-resolution component such as a vision sensor [34]. So many sensors have been developed to obtain much tactile information [21]. They used an array form to specify the contact location and predicted slip direction and speed using multi-modal sensors. However, acquiring all the tactile information requires a large and expensive sensor such as bio-tac [35]. The primary purpose of the slip sensor is to prevent slip. If the robot knows the slip speed and direction, it can be possible to prevent the slip more precisely. However, by just knowing whether or not there is a slip, it is possible to perform a successful slip detection by adding a gripping force [6,36]. In other words, even if the speed or direction at which the slip occurs is not known, if it is only possible to determine whether it is slip, it can serve as a slip sensor.

Slip can be detected by using only the data of one cell among the array sensor data [37]. In this respect, the structure of a non-array sensor that does not have distributed data and has the same sensitivity for all sensor surfaces is suitable for a slip detection sensor. The non-array sensor is simple to manufacture and has the advantage of having an omnidirectional structure. It is also easy to change the size by expanding the simple layer structure. Even if a part of a sensor is cut out, there is no problem in the operation of the remaining part. An array sensor has complicated wiring, and when some of the wires are damaged, many of the cells connected to it are not available. Since the number of signals output from the non-array sensor is little, the number of wires is also little. Moreover, the burden of the processing device can be reduced because there are fewer signals to be processed.

### 2.3. Fabrication

**Figure 2:**
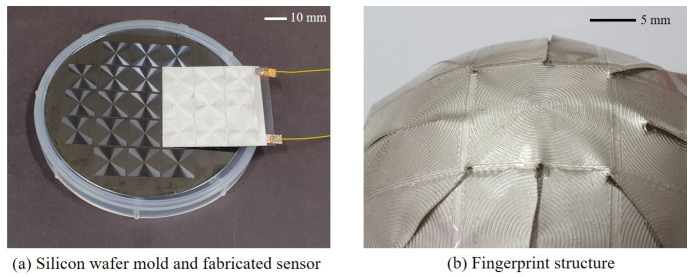
Wafer mold and fabricated sensor.

In order to manufacture a sensor that fits the proposed working principle, the following process was performed. Polydimethylsiloxane (PDMS) was used to fabricate the fingerprint structure. PDMS is a silicone rubber-based material, and it is good to make a shape using a mold. It has relatively high stiffness, so it is suitable when a rigid structure such as a fingerprint structure is required [38]. PDMS part A and part B (Sylgard 184, Dow Corning) mixed in a 10:1 weight ratio and steered for five minutes. A mold was made with a silicon wafer to shape the PDMS into a fingerprint structure, as shown in Figure 2a. The mold was made using lithography and covered with a Teflon coating for easy detachment. PDMS was poured onto the mold using spin coating so that the fingerprint structure had the same thickness. Spin coating was performed for 5 min at 300 RPM. After spin coating, air bubbles inside the PDMS were removed for 10 min using a vacuum chamber. PDMS placed on the mold was crosslinked in an oven at 120 degrees for 20 min. As a result, the PDMS was molded to have a thickness of 0.7 mm, including the fingerprint structure. The ridge of the fingerprint structure has a width of 0.2 mm, a height of 0.2 mm, and a spacing of 0.2 mm, which is similar to that of a human fingerprint (see Figure 2b) [39]. Nine fingerprint structures in the form of concentric circles were arranged to obtain a similar response, regardless of slip direction when the slip occurs.

In order to manufacture the simplest type of sensor, the sensor was manufactured with only minimal elements. A piezoelectric film was used to measure the vibration generated on the sensor surface. The piezoelectric film used at the sensor is a polyvinylidene fluoride (PVDF) material. PVDF can be shaped into various sizes and is inexpensive [28]. It is also a flexible material and is suitable for the material of tactile skin. Electrodes are formed on both sides of PVDF to transmit piezoelectric signals. In this paper, a metalized piezo film sheet (2-1004347-0, Measurement Specialties, Hampton, VA, USA) was used. This film has a 52 μm-thick PVDF layer polarized in the direction of thickness and has electrodes formed on both sides of each 6 μm-thick silver ink. The piezo film cut according to the size of the sensor was wired using copper tape. The fabricated PDMS fingerprint structure was attached to the PVDF film. The PDMS structure protects the exposed electrodes and amplifies vibrations caused by slip. As a result, a very simple structure of the slip sensor was manufactured.

### 2.4. Application to an Arbitrary Surface

For the tactile sensor to function correctly, it must be attached to the surface of the robot. When the surface is flat, it is easy to attach any tactile sensor. Nevertheless, it becomes a problem when it has curvature. Generally, three methods are used to solve this problem. One is to use a sensor made of a material that is flexible and stretchable [40]. If the curvature is not too large, the sensor can be attached to a curved surface using its elasticity. Nagakubo et al. developed a tactile distribution sensor that can be attached to a complex curved surface using a conductive rubber sheet [41]. However, this approach leads to non-uniform sensor initial values. To solve this discrepancy, a calibration process for the surface to be attached is required. Another way is to make the sensors modular and connect them with flexible wires between each of modules [42]. Since each module is placed close to the plane, the sensor manufactured in this way can exhibit stable performance. However, it cannot be attached to a curved surface below a specific curvature and makes the surface angled.

The final method is to manufacture the spot-on sensor to the surface to be attached. This method is ideally the best way to attach the sensor. However, most of the sensors use the method of manufacturing based on flat plane [5]. Manufacturing to fit a curved or spherical surface entails material limitations and complex processes [43]. Instead of the ideal method, an alternative method can be used. It is to fabricate the sensor in a flat surface and machine it to fit the surface to which it is attached [44,45]. This approach is similar to making the sensor modular. However, the difference is that each module is manufactured flexibly and has a kind of free placement. This method is an extension of the modular sensor and has a limitation that it can be attached to a curved surface within the initially designed form.

In this paper, we propose a novel sensor that can cover an arbitrary surface. The manufactured slip detection sensor has a non-array structure. A sensor with a non-array structure is like a number of sensors of the same structure connected in parallel. It is because the cross-section of the sensor is the same. As each of the sensors made in parallel is driven, a cut part of a non-arrayed sensor can be driven on the same principle. Moreover, the voltage of the piezoelectric effect is determined regardless of the area. It is determined by the applied force and the thickness of the piezoelectric material. Although the output current varies depending on the area, the current can be supplied through an external amplifier, such as a voltage follower. Thanks to this structure, even if a part of the sensor is removed, the remaining part can be operated without a hitch. Therefore, it is possible to attach the proposed sensor to any curved surface. For sensors developed in the past, the developer is often the user of the sensor. So, the sensors they make are designed for the surface they want to use. However, if the tactile sensor becomes familiar, the developer and the user are inevitably separated. The proposed sensor is very simple, and any user can freely cut-and-attach for any surface. It is an end-user friendly design [46]. It is only necessary to connect two wires to the processing unit for the sensor to work.

## 3. Slip Detection Algorithm

This section describes how to detect slip from the data of the slip sensor. Signal processing must be performed to detect slip from the vibrotactile data collected from the sensor. Feature extraction was performed to detect slip using a neural network. By the feature extraction, the sensor data were converted into an image which has time-frequency domain data. The slip was judged with four different ANN models using the extracted feature.

### 3.1. Feature Extraction

Appropriate feature extraction is critical to design a neural network with given data [47]. Frequency-domain data and time-domain data are meaningful information that can be obtained from a one-dimensional vibration signal. A spectrogram can be used to use both frequency domain and time domain data. Spectrograms have been used for speech recognition from long ago to recent day [48,49]. Since voice recognition uses vibration data of sound, it has similarities to slip detection using vibrotactile data. One of the slip detection sensors developed in the early days used a spectrogram [50]. However, as more recent slip detection sensors use arrays, the use of spectrograms has decreased. Nevertheless, the spectrogram has many useful points when analyzing vibration signals. The most significant advantage is converting 1D data into meaningful 2D data. Neural networks such as CNN can recognize the relationship between adjacent data. When using such neural networks, using more than 2D data gives a significant advantage in performance.

**Figure 3:**
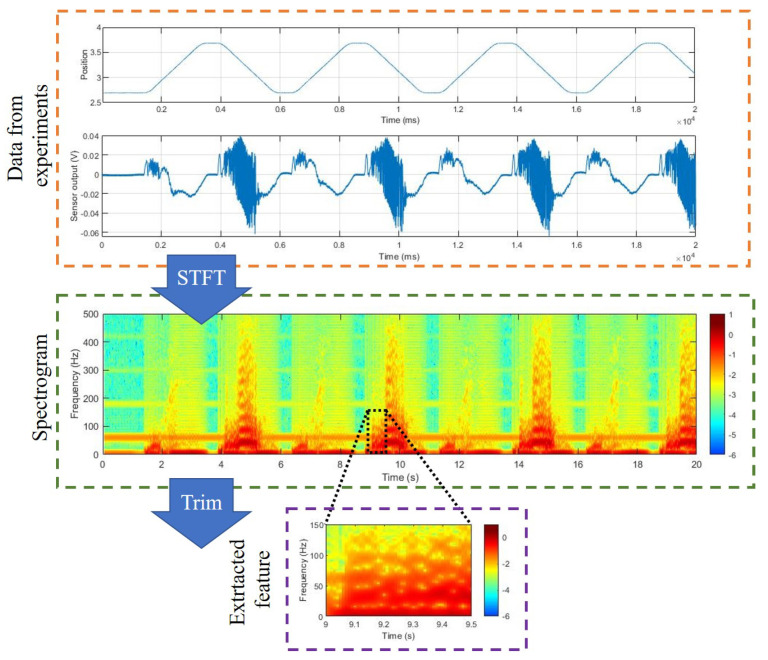
Extracted feature.

In this paper, the spectrogram is used as an input to the neural network models. The spectrogram was generated using a short-time Fourier transform (STFT). Moreover, 1024-point fast Fourier transform (FFT) was performed using tactile data sampled at 1 kHz. It is suitable for dexterous manipulation to detect and respond to slip in a short time. Therefore, to minimize the time delay, the window size and overlap size were applied as 100 and 90. Data were windowed by the Hamming window. Part of the spectrogram was used as a feature to determine the slip. If too large data are used, the burden on the neural networks becomes too great. Data of 0–150 Hz in the frequency domain and 0.5 s in the time domain were used to determine the slip. Through this process, features are extracted, like Figure 3.

### 3.2. Neural Network Model

When the spectrogram is generated through the STFT, 2D data having information in the frequency domain and the time domain can be obtained. It is inaccurate to judge whether or not to slip simply by using a magnitude of a specific frequency band. It is because the frequency of vibration generated by slip varies depending on the vertical force in contact, the slip speed, and the frictional force of the contact surface. In order to more accurately determine slip, changes in frequency over time should be observed. The neural networks are very useful tools when deriving the desired information using such a large amount of data.

Various types of ANNs have been developed. Depending on which model is used, the slip detection performance may vary [7]. Feedforward neural networks (FNN), primary neural networks, have an entirely connected neuron structure from input units to output units. Since all input data are given as a one-dimensional vector, the positional correlation between them is ignored. Neural networks that overcome these disadvantages are CNN [51]. The unit of CNN outputs a convolution of a set of a specific size from the input data. Through this, it is possible to transfer the relationship between data close to each other to the next layer. CNN consisting of several layers has powerful performance for image analysis. ANNs that include temporal correlation instead of spatial correlation are recurrent neural networks (RNN) [52]. The RNN can transmit information according to the passage of time to the next source by circulating data from the previous time to the current time. The basic RNN only receives data from the previous time, so data older than the previous ones are indirectly received. As a result of this data flow, there is a problem that the amount of information received from old data decreases. LSTM is a model that improves these shortcomings. LSTM has gates, which play a role in deciding whether to forget or keep remembering information about the previous time. Even old data can be used without damage if the gate keeps remembering meaningful data [53]. ConvLSTM can be viewed as a combined CNN and LSTM model [54]. While the existing LSTM was designed for neurons based on ANN, the ConvLSTM was designed for neurons based on CNN. Through this, data can be determined using both spatial correlation and positional correlation. In this paper, several neural network models compare which model is more advantageous for slip detection. There are four neural networks used as follows: CNN, LSTM, CNN-LSTM parallel combination, ConvLSTM.

**Figure 4:**
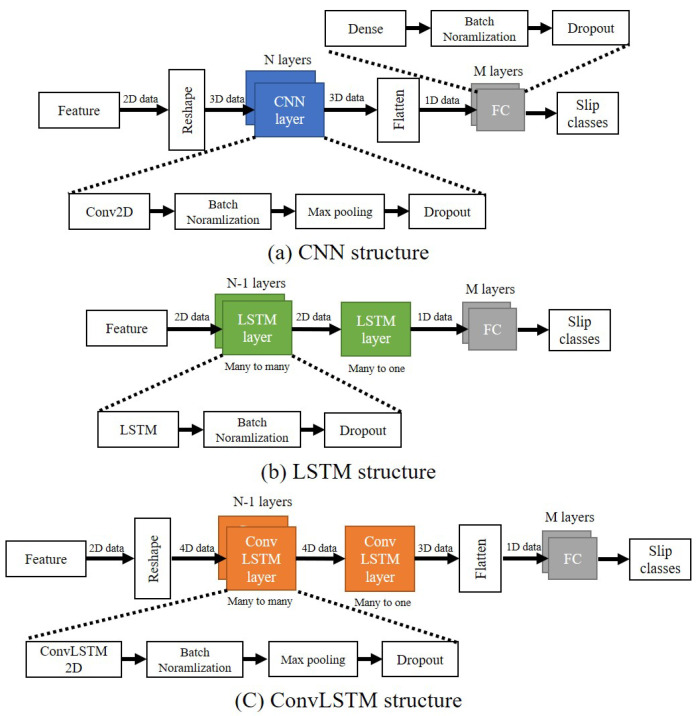
Neural networks structure.

All neural network models use features generated through STFT as input. The feature is used by reshaping it to the input dimension suitable for each model. Each output of the model is converted into a one-dimensional array and then inputted to the fully connected (FC) layer. The last FC layer, which is the output layer, finally outputs whether or not the object slips. This process is as Figure 4. In all models, the batch normalization momentum was 0.99. The batch size was 128. Adam and accuracy were used as the optimizer and metric, respectively. For the dropout rate and learning rate, optimal values were used through the grid search in each model. The results of the grid search are described in Section 4. The activation function and initializer of each neural network are shown in the Table 1.

CNN uses 3D data, a set of 2D images, as input. Another dimension with a value of 1 was added to input a feature that is 2D data. Through this, a feature matrix with a size of 50 × 150 becomes a matrix with a size of 50 × 150 × 1. After that, it passes through the CNN layers composed of N layers. Each CNN layer is composed of Conv2D (3 × 3 filter window size), batch normalization, max pooling, and dropout layers (see Figure 4a). Data passed through the CNN layers are converted into a one-dimensional vector to input the FC layer. Each of the M FC layers is composed of dense, batch normalization, and dropout layers (see Figure 4a). N and M and the number of units of each layer were determined through grid search. At the last FC layer, binary classification is performed to determine whether or not to slip.

LSTM uses 2D data features as input. LSTM layers consist of N layers. Each LSTM layer is composed of LSTM, batch normalization, and dropout layer (see Figure 4b). N-1 LSTM layers are many to many layers and receive 2D data as input and output 2D data. The last one LSTM layer is many to one layer, and it receives 2D data as an input and outputs 1D data. The output 1D data passes through FC layers composed of M layers. The rest configuration is the same as the CNN structure.

The CNN-LSTM parallel model uses CNN and LSTM layers in parallel. This model was designed based on the architecture proposed by Bae et al. [58]. Features are duplicated and passed through each layer, then concatenates and input to FC. The composition of the CNN, LSTM, and FC layers is the same as the previous two structures.

ConvLSTM uses 4D data as input. Two more dimensions with a value of 1 were added to input a feature that is 2D data. Through this, a feature matrix with a size of 50 × 150 becomes a matrix with a size of 50 × 1 × 150 × 1. After that, it passes through the ConvLSTM layers composed of N layers. Each ConvLSTM layer consists of ConvLSTM2D (3 × 1 filter window size), batch normalization, max pooling, and dropout layers (see Figure 4c). Data passing through the ConvLSTM layers are converted into a one-dimensional vector for input to the FC layer. The rest of the configuration is the same as the CNN structure.

## 4. Experiments and Result

In this section, experiments are conducted to verify the performance of the sensor, and the results are presented. In order to operate the slip sensor, the ANN must be learned first. Besides, each of the ANN models needs to be optimized to realize high slip detection performance. It consisted of three experiments as follows.

In the first experiment, a sensor was attached to a plane and slipped. This experiment is performed to optimize the ANN model. Different directions of slip at different points on the sensor surface were generated, and the slip detection accuracy at this time was compared. For optimizing each ANN model, the number of hidden layers, the number of units in the hidden layer, and the dropout rate and learning rate were optimized.

In the second experiment, the sensor was attached to several curved surfaces with different curvatures and slipped. This experiment serves two purposes. First, it checks whether the sensor can be used even if the sensor is attached to a curved surface with different curvature. Second, it collects training data to use the sensor on the sphere surface. Through the experiment, we compared the accuracy of the slip detection on each curved surface. In the third experiment, the sensor was attached to several spheric surfaces with different curvatures and slipped. Through this experiment, we can check whether the sensor can be driven on any surface.

We sampled at 1 kHz using a DAQ device (PXI 4472B, NI) to measure the response of the sensor. The output of the sensor was transformed into STFT by MATLAB R2019b. The specifications of the computer used in the experiment are described below: CPU Intel(R) Xeon(R) E5-2630 v4; RAM: 128 GB DDR4; GPU: Nvidia TITAN RTX 24GB; SSD: NVMe Samsung SSD 960 500 GB; OS: Windows 10 64-bit. All neural networks were trained with the TensorFlow 2.1 framework on Python 3.7.

### 4.1. Flat Surface and Model Optimization

**Figure 5:**
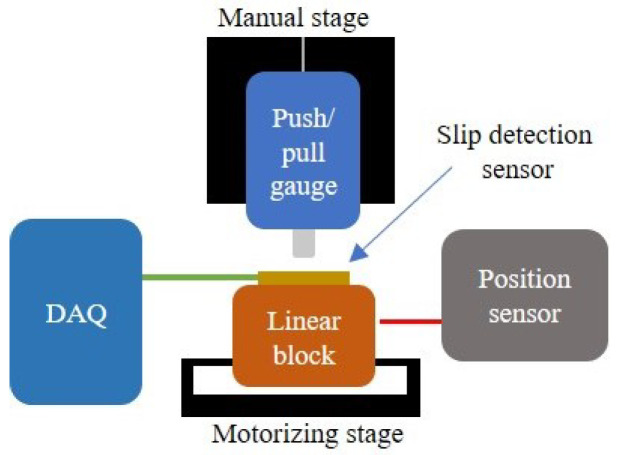
Testbed for slip experiment.

First, to optimize the artificial neural network, an experiment was performed by attaching a slip sensor to a flat surface. The sensor attached to the plane generates slip through slip test equipment as Figure 5. The sensor and the flat surface are fixed to the motorizing stage, moving left and right. A vertical manual stage was used to press the object against the sensor surface with a constant force. With a constant force applied to the sensor, the motorizing stage is driven to generate a forced slip on the sensor surface. This simulates slip occurrence due to external factors when the robot grips an object with a constant force. The signal generated at this time is collected through DAQ. Moreover, the position of the motorizing stage is collected through a position sensor. The signal collected from the position sensor is used to generate a label to determine whether it is slipping.

When slip is generated using a motorizing stage, a similar vibration signal can be repeatedly generated. It may have a problem of deteriorating accuracy in a practical environment by overfitting a specific pattern. However, when comparing several experiments in which some variables were changed, a consistent response can be expected. Because we compare and use several neural network models, we need a consistent response. Due to this point, repeated experiments were performed.

**Figure 6:**
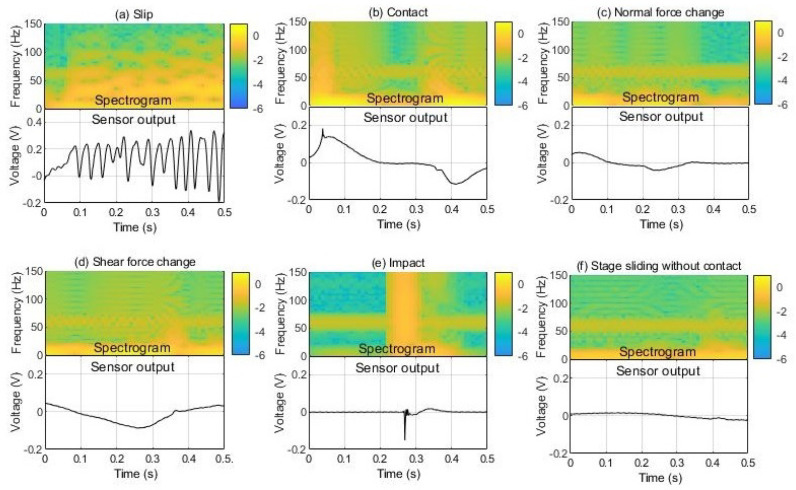
Slip and nonslip training data.

To determine whether or not to slip with the vibration signal, training of the ANN is essential. In order to learn whether to slip or not, data with and without slip are required. At this time, the non-slip data can be confused with the slip, but it should be data that does not slip. Otherwise, the learning process seems to distinguish the slip well, but it will not in the practical environment. In order to produce the training data, the following slip data and non-slip data were composed as Figure 6. The slip data were produced by varying the vertical force and slip speed to detect the slip in various environments. The normal force was applied with three forces of 0.2, 0.4, and 0.6 N, and the slip speed was generated at three speeds of 15, 30, and 45 mm/s. Non-slip data gave the following five inputs that could be confused with slipping. These five inputs are contact-detachment, normal force change, shear force change, impact, and stage sliding without contact. Uncontact stage sliding is the operation of the motorizing stage when nothing is in contact with the sensor. It was selected to add the vibration factor of the motor to the training data.

**Figure 7:**
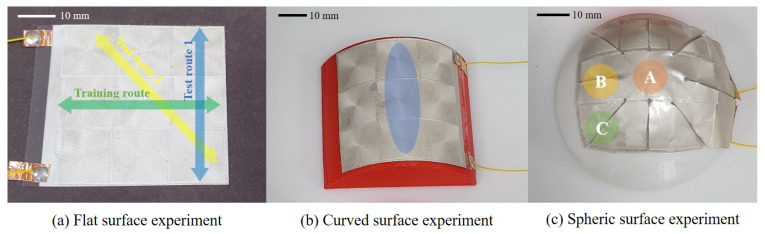
Contact point of each experiment.

A training set and a separate test set are required to check the slip detection performance on ANN. The test set may be configured by separating a part of the training set or being configured by additional experimenting. When using a testbed like Figure 5, very similar responses can be repeatedly obtained due to the motorizing stage. As a result, unrealistic accuracy of over 99.99% occurs when using k-fold cross-validation. These abnormal results make it impossible to perform proper model evaluation and optimization. For a more accurate evaluation, a separate experiment was used to create a test set. The test set was produced by giving a slip in a different direction and a different position than training data. The developed slip sensor was proposed to detect slip on all sensor surfaces. Therefore, the ability to learn the slip occurring in a part of the surface and detect the slip occurring in another part is reasonable. For this, a training set was constructed with the slip of Figure 7a: Training route. The test set was composed of the slip of Figure 7a: Test routes 1, 2 that are separated from the training set. Test route one was chosen to use a slip in a different direction from the training set. Test route two was selected to use a slip that deflected the center of the fingerprint structure.

**Figure 8:**
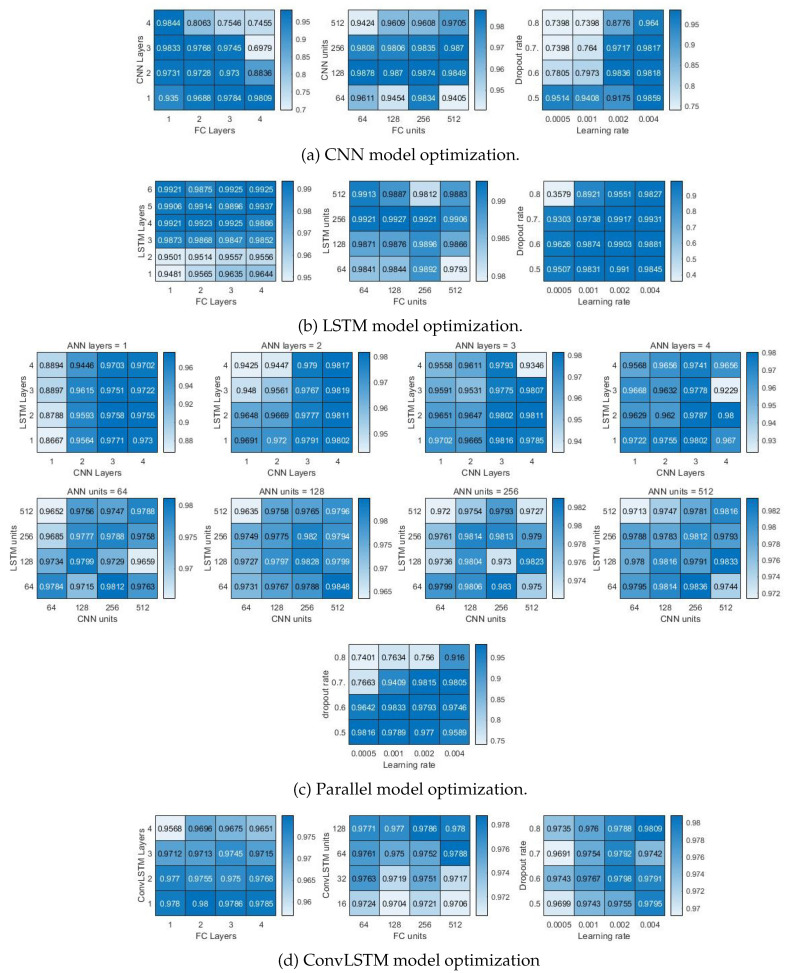
Neural networks models—grid search result.

The ANN models were trained with the training set obtained through the experiment. Although the accuracy can be improved through learning, the hyperparameters do not change with learning. Therefore, model optimization is required to create a high-performance ANNs. As the hyperparameter value, such as the number of hidden layers, increases, the neural network can determine more complex data. However, a too large model may overfit the training data, resulting in poor performance in actual test data. It is imperative to select hyperparameters with appropriate values to achieve an optimal model. Grid search is the process of finding the optimal model by changing the hyperparameter. The hidden layer, unit, dropout rate, and learning rate of the neural network model were optimized using grid search as Figure 8.

**Figure 9:**
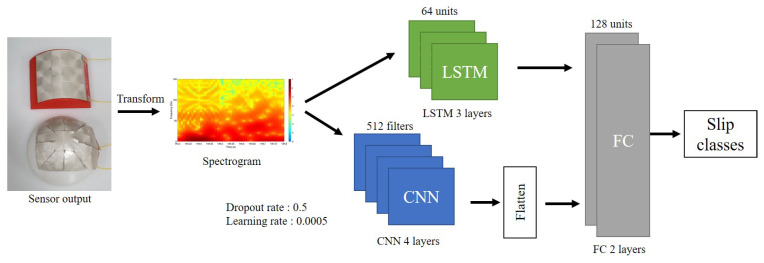
Parallel model—diagram.

Among the four ANN models, the optimization process of the CNN-LSTM parallel model is as Figure 8c. The parallel model consists of a CNN layer and an LSMT layer connected in parallel, and an FC layer. The grid search was performed with different numbers of hidden layers for these three structures. The accuracy was compared for a total of 64 models with a different number of the hidden layer. CNN, LSTM, and FC have four different sizes of hidden layers, from 1 hidden layer to 4 hidden layers, respectively. In consideration of the influence on the random initial value, ten training was performed, and each model was compared using the accuracy of the average value. As a result, a model with the LSTM 3 layer, CNN 4 layer, and FC 2 layer showed the highest accuracy. The same process was applied to the number of units in the hidden layer. Furthermore, 64, 128, 256, and 512 units were selected as the number of units. Accuracy was compared for 64 models created through this process. As a result, the model using 64 units of LSTM, 512 of filters with CNN, and 128 units of FC showed the highest accuracy. Moreover, the grid search was performed for the dropout rate and learning rate. Dropout rate was used for 4 values of 0.5, 0.6, 0.7, 0.8, and learning rate was used for 4 values of 0.0005, 0.001, 0.002, and 0.004. The best results were obtained when the dropout rate was 0.5, and the learning rate was 0.0005. As a result, the structure of the optimized CNN-LSTM parallel model appeared as shown in the Figure 9. The same process was carried out for the remaining 3 ANNs, and the results are shown in Table 2.

### 4.2. Curved Surface

**Figure 10:**
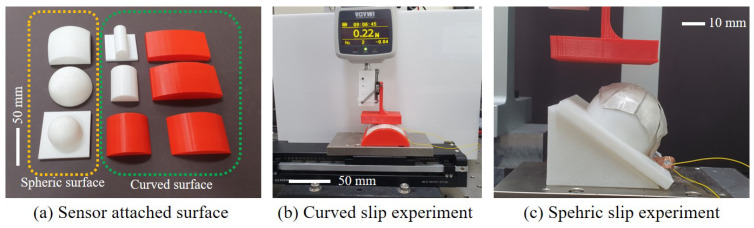
Curved and spheric experiment.

Through the optimization process, four ANN models to be used for slip detection were prepared. The next experiment was conducted by attaching a sensor to a curved surface to compare slip detection performance among each model. Sensors were attached to six different curved surfaces to check whether the sensor can work well on various curved surfaces. The radius curvature of each surface is 200, 100, 50, 30, 20, 10 mm as Figure 10a. Slip occurred in blue area in Figure 7b. The configuration of the training set and the test set is the same as that of the flat surface. The testbed where the experiment was performed is the same as the previous experiment. However, the sensor was attached to the curved surface, as shown in Figure 10b. A tip was manufactured in a form similar to a stamp to generate slip on the curved surface. This tip applies a constant force to the sensor continuously while slip occurs.

Neural networks perform better as they learn with more data. However, collecting much data increases the cost. Different training data were used in three steps to check whether the model performance differs depending on the data amount. First, the slip data at a flat surface was used as the training data. Second, the neural network was trained using all the data of flat and curved surfaces. However, the data of the test surface were not included in the training set. The reason for excluding data of test curvature is to separate the training set and the test set. Finally, data on the flat surface and the 10 mm curved surface, which is the smallest curvature, were used as training data. The third training data was constructed to check the middle of the first and the second training data, as the training data was minimal in the first case, and the training data was extensive in the second case.

**Figure 11:**
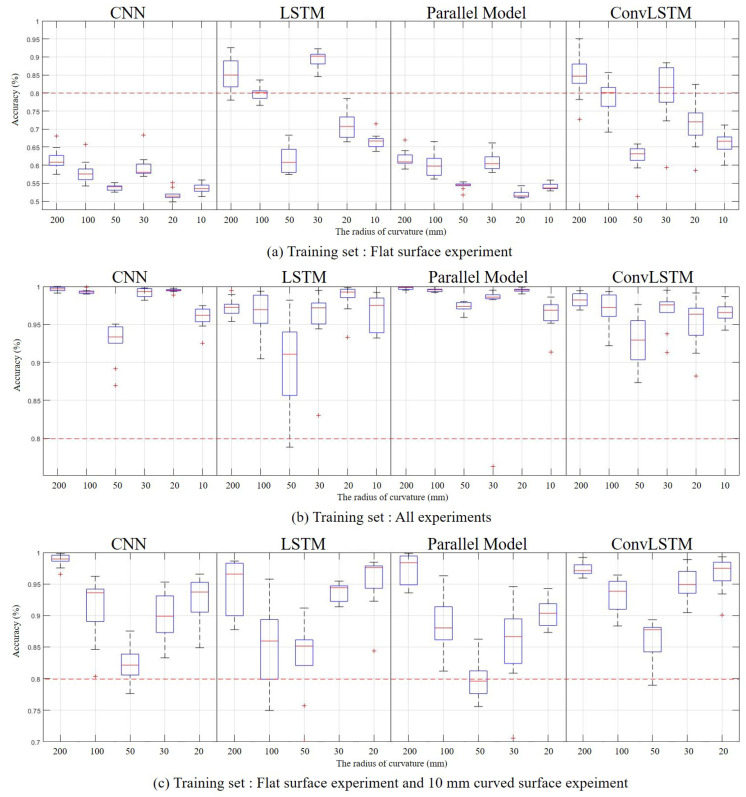
Result: Curves surface experiment.

First, training was performed using only the slip data on a flat surface, and then the slip data for each of the six curved surfaces was classified. Initialization and training were performed for each of the four models ten times, and accuracy was compared. The accuracy of 6 curved surfaces for four models is shown as a box graph in Figure 11a. The top of the box in the box graph is the top 75%. The bottom of the box is the bottom 25%. The middle red line is the median value, the upper part of the beard is the maximum value, and the lower part is the minimum value. The red crosshair separated from the box is an outlier. In general, it can be seen that the smaller the curvature, the lower the accuracy. The CNN and the CNN-LSTM parallel model showed an accuracy of less than 70% for all six surfaces. This learning state cannot be used because of poor accuracy in binary classification. LSTM and ConvLSTM show slightly higher accuracy than the CNN and the parallel model. The curved surface with a curvature of 200 mm shows moderate accuracy. However, the average accuracy is still low, and when the curvature is low, the accuracy is less than 70%. The average accuracy of CNN is 56.4%, LSTM is 75.6%, parallel model is 57.1%, and ConvLSTM is 73.9%. When these results are put together, using neural networks trained with flat surface data shows insufficient accuracy.

Second, after training using the slip data for all surfaces, excluding the test surface, the slip data for each of the six surfaces were classified as Figure 11b. As a result, it was possible to obtain reasonably high accuracy in all four models. The CNN and the parallel model, which showed low accuracy in the first training, showed very high accuracy this time. In particular, the parallel model shows an accuracy of more than 95% for all curved surfaces, and it can be seen that the slip can be detected with high performance. LSTM and ConvLSTM show a large deviation in accuracy between training compared to the other two models. It showed very high accuracy in some training, but very low accuracy in other training. It means that the learning result does not have stable training accuracy in the realistic environment, and there is a problem that the reliability is degraded. The average accuracy of CNN was 97.6%, LSTM was 95.7%, the parallel model was 98.1%, and ConvLSTM was 96.1%.

The third training result is to see the median value of the previous two training results. Slip data for a flat and a 10 mm curved surface were used as training data. As test data, slip data for five curved surfaces with radius curvatures of 200, 100, 50, 30, and 20 mm were used. As a result, similar levels of accuracy were confirmed for all four models. In most cases, it shows an accuracy of 80% or more, and it is possible to train the model to a usable level. The higher accuracy was obtained in the environment similar to the training surface since the training was performed with the data of the flat and the curvature of 10 mm. The other way, the lower accuracy was obtained in a less similar environment. As a result, according to the curvature, the distribution of accuracy was shown as a V-shaped curve. In most situations, it showed that the accuracy deviation for training was large. The average accuracy of CNN was 90.9%, LSTM was 90.5%, the parallel model was 88.4%, and ConvLSTM was 93.7%.

The first training showed low accuracy using extremely little training data. It can be seen that if the training data are too few, the accuracy can be lowered to an unusable degree. If the data is small, the cost required for learning would be reduced, but the slip sensor needs enough data to operate normally. The second training showed very high accuracy using quite a large amount of training data. This indicates that even a very simple sensor can determine the slip well when enough data is provided to the ANN. However, in order to prepare an extensive training data, it may take more cost to prepare. In the third training, we used the compromised training data of the above two pieces of training. As a result, it used 66.6% less data than the second training and showed a 72.2% lower error rate than the first training. So, it is possible to training to the usable state. Moreover, slip detection accuracy can be improved using data on curved surfaces with different curvatures. It indicates that when there are sufficient data, the slip can be detected on any curved surface. Therefore, the sensor can operate with the same principle, although the curvature of the surface affects the accuracy.

The CNN and the CNN-LSTM parallel model showed similar trends according to the training data, and the LSTM and the ConvLSTM model showed similar trends. Because the CNN-LSTM model is similar to CNN, and the ConvLSTM model is made similar to LSTM. The CNN-LSTM parallel model consisted of 4 CNN hidden layers with 512 filters and 3 LSTM hidden layers with 64 units. In other words, since there are many more units used in the CNN, it works dominantly on the CNN. The ConvLSTM model processes data using a convolution filter, but in the end, it is a kind of RNN and is close to LSTM. The former models showed relatively high accuracy with more data (second training), and the latter models with less data (first training) showed relatively high accuracy. In the third training, where the amount of data is moderate, the four models showed similar accuracy. Through this, it can be seen that the fewer data used for training, the more advantageous the LSTM and the ConvLSTM model, and the more data used for training, the more advantageous the CNN and the CNN-LSTM parallel model.

### 4.3. Spheric Surface

A curved surface experiment found that the sensor operates on the same principle for curved surfaces with different curvatures. The sensor was attached to the spheric surface to see if the same principle applies to further worse conditions. Attachment to the spheric surface is to check whether the sensor can be used after cutting and attaching a part of the sensor to any surface. If the sensor has sufficient accuracy for a spheric surface, it can be applied to any surface. The training data were prepared for surfaces of 50, 30, and 20 mm curvature. The slip was generated at three points for each spheric surface to check whether the slip was well sensed for the cut part. In Figure 7c, Area A is the central part of the spheric surface and is the part that is minimally affected by the cut. Area B is the portion of the cut that is side to the center. Area C is where the cut was made in the diagonal direction, the most cut was made, and the part that was farthest from the center. The spheric surface was placed on the slope to generate slip at these points, as Figure 10c.

**Figure 12:**
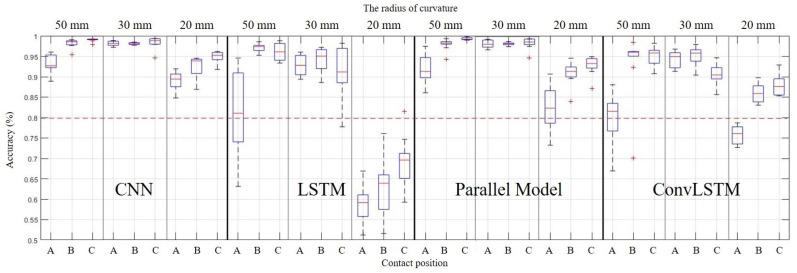
Result 2.

The curved surface data produced in the previous experiment were used as training data to classify the slip. The training was performed on all the data generated on the flat and the curved surface. The accuracy of the slip detection on the spheric surface was shown in Figure 12. As a result, the CNN and the parallel model showed quite a high accuracy. Initially, it was expected that the center portion, which was not affected by the cut, had high accuracy, and the diagonal portion that was affected by the cut had low accuracy. However, as a result of the experiment, the results were opposite to those expected. It seems that the effect between the sensor and the attachment surface was greater than that of the cutting. This is because the further away from the cut part, the greater the curvature difference between the sensor and the attachment surface. As a result, the slip could be detected with sufficiently high accuracy on the spheric surface. However, it showed remarkably low accuracy in the central part(area A on Figure 7c), and transfer learning was performed to improve this.

Transfer learning is a technique for obtaining high accuracy with little data by additional learning a part of the model for a model that has already been learned. Additional spheric data were trained for transfer learning in the model trained with a large amount of curved surface data. Spheric surface data to be trained were additionally produced to be differentiated from test data. Training data for transfer learning were produced on a smaller scale than the existing training set to check the performance when adding data at a minimal cost. As for the existing training data, slip data were produced for three forces and three slip speeds. In contrast, the data for transfer learning were produced for one force of 0.4 N, and one slip speed of 30 mm/s. It is assumed that transfer learning is implemented at the minimum cost by using little data compared to the existing training data. Furthermore, data were produced only for the central part of the spheric surface showing the lowest accuracy.

**Figure 13:**
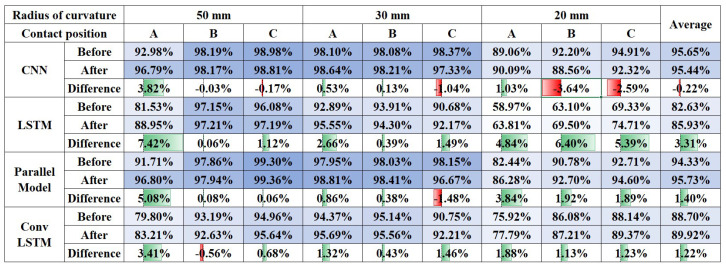
Comparison of accuracy before and after transfer learning.

As a result, there was much improvement in accuracy for the central area as Figure 13. In particular, an apparent increase in accuracy has occurred in the LSTM model, which showed high accuracy with little data. However, in the CNN model that requires many data for high accuracy, the overall accuracy decreases. This problem occurred because data on the side and diagonal parts were not in the transfer learning data. The CNN-LSTM parallel model overcomes these shortcomings of CNN. Due to the influence of the LSTM included in the parallel model, it was possible to improve the accuracy of the center part considerably, while also increasing the accuracy of the rest part. Through this, the CNN model showed the best overall accuracy before transfer learning, but the CNN-LSTM parallel model showed the highest overall accuracy after transfer learning.

## 5. Conclusions and Future Work

In this paper, we developed a slip detection sensor manufactured with a simple structure and can easily change its shape. The developed sensor has a simple structure combining PVDF and PDMS fingerprint structure and can be produced at a low cost. The sensor structure is designed in a non-array structure. So it can be used even if a part of the sensor is cut. Thanks to these characteristics, the user can tailor the shape of the sensor directly to the surface to which the sensor is attached. Moreover, even if the surface has an arbitrary curvature, the sensor can be attached.

The vibration signal from the sensor was converted into a spectrogram and used as an input to a neural network. Four neural network models were used to compare the slip detection performance in various ANNs. The used models are CNN, LSTM, CNN-LSTM parallel model, and ConvLSTM model. In order to derive the maximum performance of each neural network, optimization was performed for each model. The number of hidden layers, the number of units, the dropout rate, and the learning rate were selected as optimization parameters. In the selected parameter, the optimal value was found using a grid search.

For the four tuned models, the slip detection performance on curved and spherical surfaces was compared. Each model showed different performance depending on the training data. CNN and parallel model showed superior performance when there were many training data. LSTM and ConvLSTM showed superior performance when the training data were few. When training data were given at an intermediate level, CNN showed the best performance with an average of 90.85%. When the slip was detected on the spheric surface, the training was performed using all the curved surface slip data. Thanks to that, the CNN and parallel models showed superior performance over LSTM and ConvLSTM. The CNN model showed the best performance, with an average of 95.44%. Transfer learning was performed to improve the disadvantage of lowering accuracy when contact with the sensor changes. As a result of transfer learning, the parallel model showed the highest accuracy at 95.73%.

These results confirmed that when a sensor is attached to a spheric surface, the accuracy of slip detection varies depending on the contact position. In future studies, we will aim for a training process that can improve performance after attaching a sensor.

## Figures and Tables

**Table 1:** Activation function and initializer of each neural network.

	CNN	LSTM	ConvLSTM	FC	Output Layer
Activation function	Relu [55]	Hyperbolic tangent	Hyperbolic tangent	Relu	Sigmoid
Initializer	He uniform [56]	Glorot uniform [57]	He uniform	He uniform	Glorot uniform

**Table 2:** Result of the grid search.

Neural Network Structure	CNN	LSTM	Parallel	ConvLSTM
The number of hidden layer of CNN/LSTM/ConvLSTM	4	4	CNN: 4 LSTM: 3	1
The number of hidden layer of FC	1	3	2	2
The number of units of CNN/LSTM/ConvLSTM	128	256	CNN: 512 LSTM: 64	64
The number of units of FC	64	128	128	512
Dropout rate	0.5	0.7	0.5	0.8
Learning rate	0.004	0.004	0.0005	0.004

## Abbreviations

The following abbreviations are used in this manuscript:

ANN	Artificial neural networks
FBG	Fiber Bragg grating
CNN	Convolutional neural networks
LSTM	Long short-term memory
DNN	Deep neural networks
PDMS	Polydimethylsiloxane
PVDF	Polyvinylidene fluoride
STFT	Short-time Fourier transform
FFT	Fast Fourier transform
FNN	Feedforward neural networks
RNN	Recurrent neural networks
FC	Fully connected
